# Reduction strategies for hierarchical multi-label classification in protein function prediction

**DOI:** 10.1186/s12859-016-1232-1

**Published:** 2016-09-15

**Authors:** Ricardo Cerri, Rodrigo C. Barros, André C. P. L. F. de Carvalho, Yaochu Jin

**Affiliations:** 1 Department of Computer Science, UFSCar Federal University of São Carlos, Rodovia Washington Luís, Km 235, São Carlos, 13565-905 SP Brazil; 2 Faculdade de Informática, Pontifícia Universidade Católica do Rio Grande do Sul, Av. Ipiranga, 6681, Porto Alegre, 90619-900 RS Brazil; 3 Instituto de Ciências Matemáticas e de Computação, Universidade de São Paulo, Campus de São Carlos 135, São Carlos, 13566-590 SP Brazil; 4 Department of Computer Science, University of Surrey, GU2 7XH Guildford, Surrey, United Kingdom

**Keywords:** Hierarchical multi-label classification, Protein function prediction, Machine learning, Neural networks

## Abstract

**Background:**

Hierarchical Multi-Label Classification is a classification task where the classes to be predicted are hierarchically organized. Each instance can be assigned to classes belonging to more than one path in the hierarchy. This scenario is typically found in protein function prediction, considering that each protein may perform many functions, which can be further specialized into sub-functions. We present a new hierarchical multi-label classification method based on multiple neural networks for the task of protein function prediction. A set of neural networks are incrementally training, each being responsible for the prediction of the classes belonging to a given level.

**Results:**

The method proposed here is an extension of our previous work. Here we use the neural network output of a level to complement the feature vectors used as input to train the neural network in the next level. We experimentally compare this novel method with several other reduction strategies, showing that it obtains the best predictive performance. Empirical results also show that the proposed method achieves better or comparable predictive performance when compared with state-of-the-art methods for hierarchical multi-label classification in the context of protein function prediction.

**Conclusions:**

The experiments showed that using the output in one level as input to the next level contributed to better classification results. We believe the method was able to learn the relationships between the protein functions during training, and this information was useful for classification. We also identified in which functional classes our method performed better.

## Background

In the majority of the classification tasks found in the literature, a single class (concept) is assigned to a given instance (object), and the problem classes assume a flat (non-hierarchical) structure. However, in a variety of real-world applications, classes are organized in a hierarchical structure, where they are specialized into subclasses or grouped into superclasses. These classification problems are known in the machine learning (ML) literature as hierarchical classification (HC), since instances are assigned to classes associated with nodes of a hierarchy. Depending on the domain problem, a hierarchical class structure can be represented as a tree or as a directed acyclic graph (DAG).

**Fig. 1:**
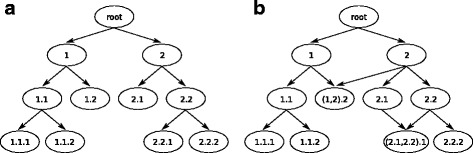
Hierarchies structured as: (**a**) trees; (**b**) DAGs. The “ ·” symbol separates classes from superclasses/subclasses (2.1 means that 2 is a superclass of 1). Adapted from [43]

In hierarchical problems with classes structured as a tree, each class node has only one parent node. In DAG structures, however, a class node can have multiple parent nodes. Therefore, in tree-structured problems, each class has a single depth value (number of edges between the root node and a given node), because there is just one possible path between the root and any other node in the hierarchy. Hierarchies structured as DAGs, on the other hand, allow for multiple depth values, since there may be more than one path between the root node and any other given hierarchical node. These characteristics should be considered in the development and evaluation of hierarchical classifiers. Figure 1 depicts hierarchies structured as either trees or DAGs.

In this paper, we investigate a particular case of HC problems in which instances can be simultaneously assigned to many classes that belong to the same hierarchical level. These problems are known as Hierarchical Multi-Label Classification (HMC), and can be formally defined as:

### **Definition**

Considering **X** the space of instances, the classification problem consists of finding a function (classifier) *f* to map each instance **x**_*i*_∈**X** to a set of classes *C*_*i*_∈*C*, with *C* the set of classes in the problem. The function *f* must respect the constraints of the hierarchy, and optimize a quality criterion.

To respect the constraints of the hierarchical structure means that when a class is predicted, all its superclasses should also be predicted. As quality criterion, one could chose to optimize que predictive performance of the classifier. This could consider, e.g., the distances between the predicted and true classes in the hierarchy, measured as the number of edges between the classes. Considering that closer classes tend to represent more similar categories, one could weight incorrect classification proportionally to the number of edges between the true and predicted classes. The classification error then tends to increase with the number of edges between classes.

The are some problems related to assigning weights to the edges of the hierarchy, specially when its depth varies significantly for different leaf nodes. When this occurs, errors involving classes near the root are less penalized than errors involving classes located deeper in the hierarchy.

In this direction, Lord et al. [1] showed that when the path connecting two classes has to go through the root node, and one of the classes is located in a deeper level than the other, this does not necessarily mean that the deeper class provides more significant information than the class located in a higher level. Thus, consider depth without considering class information may be a problem. Because of this, in this manuscript we consider only the predictive performance of the classifiers as quality criterion, without assigning weights to edges in the hierarchy.

HMC problems can be solved by either local or global approaches. Regarding local approaches, classification algorithms such as decision-tree induction or support vector machines are used to obtain a hierarchy of classifiers, which are later used to classify unlabeled instances following a top-down strategy [2]. According to Silla et al. 2010 [3], different strategies can be used in the local approach: one local classifier per node (LCN), one local classifier per parent node (LCPN), and one local classifier per level (LCL). While LCN induces one binary classifier for each class [4], LCPN induces a multi-class classifier for each parent node, in order to distinguish between its subclasses [5]. The LCL strategy induces one multi-label classifier for each hierarchical level, where each one predicts the classes of its associated level [6].

Local-based strategies can be seen as particular cases of *ML reductions*. We call reductions the techniques used to transform more complex problems into well-studied ML problems. These simpler problems are solved using any existing learning algorithm. These solutions to the simpler problems are then used to solve the original problem [7].

The global approach, differently from the local one, trains only one classifier to cope with all hierarchical classes. The classification of new instances is performed in just one step [8]. Because only one classifier is used, the specificities of the classification problem must be considered. Thus, it is not possible to use traditional classification algorithms, unless they are adapted to cope with class hierarchies.

Protein function prediction is a typical case of HMC, since protein functions are hierarchically organized. This is a very relevant classification task, since almost all functions related to cell activity are performed by proteins. They can have a great variety of forms and perform functions such as biochemical reactions, cell signaling, structural, and mechanical functions [9].

In this paper, we approach the protein function prediction HMC problem with a new reductionist method termed **H**ierarchical **M**ulti-Label **C**lassification with Local **M**ulti-**L**ayer **P**erceptrons (HMC-LMLP). A very preliminary version of HMC-LMLP has been reported in [6], where we associated one Multi-Layer Perceptron (MLP) to each hierarchical level, and used the instances as input to the MLP associated with the first hierarchical level. From the second level onwards, each MLP was fed only with the output provided by the previous MLP. Differently from the version in [6], the method proposed in this paper uses the output from the MLP trained for level *l* as part of the input of the MLP for level *l*+1. Thus, the outputs from the MLP associated with level *l* are now used to augment the feature vectors that are employed to train the MLP for level *l*+1. The idea is to guarantee that label dependencies between classes are taken into account, and also to allow the MLP classifiers to discover these dependencies by themselves.

Two other variants of HMC-LMLP, reported in [10], are considered as baseline approaches to verify whether HMC-LMLP is capable of significantly improving classification accuracy. In the first variant, the true labels of the training instances are used as part of the input to train each MLP. Therefore, when training an MLP for level *l*+1, the feature vector is augmented with its true classes for level *l*. This modification forces the label dependencies between classes to be taken into account, with these dependencies being provided by the training instances (true classes). The second variant ignores the labels associated with the classes to augment the feature vectors. This can be considered as a baseline version that allows us to examine whether the use of the labels to augment the feature vectors results in an improved classification performance.

To the best of our knowledge, our study is the first one that employs neural networks for HMC function prediction problems within the LCL strategy. A competitive neural network was proposed in [11] and applied to DAG structured hierarchies, while in [12] neural networks were used in a LCPN strategy specific for hierarchies structured as trees. In [13], stacked Extreme Learning Machines [14] neural networks were used for classification. Structured classification problems, which include hierarchical classification of protein functions, were addressed by [15] using a decision tree-based method.

**Fig. 2:**
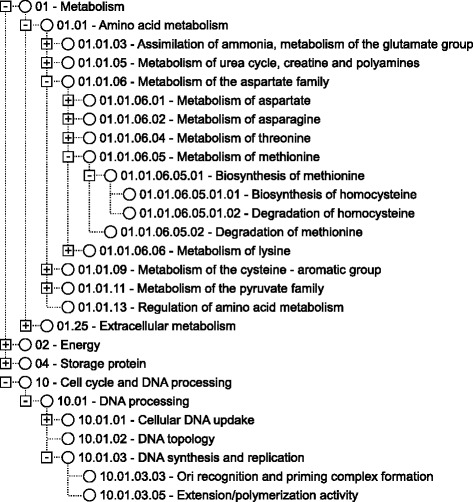
Part of the funcat hierarchical taxonomy. Adapted from http://www.helmholtz-muenchen.de/en/ ibis

Our contribution is centered in the Funcat hierarchy [16], a famous taxonomy for the functional organization of proteins of prokaryotic and eukaryotic origin. The taxonomy is a tree with up to six levels in depth, consisting of 28 main functional categories that cover functions like cellular transport, metabolism, and communication. Figure 2 illustrates a small portion of the Funcat taxonomy.

### Related work

Typically, the protein function prediction problem is solved by using homology, comparing proteins through some alignment tool, and also comparing protein folds and biding sites [17, 18]. This section discusses some recent HMC methods reported in the literature that make use of ML for protein and gene function prediction.

Vens et al. [8] investigated methods based on Predictive Clustering Trees (PCT): the global Clus-HMC method, which trains only one decision tree considering all the classes in the hierarchy, the local Clus-SC, which trains a separated decision tree for each class, and ignores the relationships between classes, and the local Clus-HSC, which also trains a decision tree for each class, but explores the relationships between them. In another study, Schietgat et al. [19] combines the Clus-HMC method using bagging.

Alves et al. [20] proposed a global method using Artificial Immune Systems (AIS) for the generation of HMC rules. The method is divided into two basic procedures: Sequential Covering (SC) and Rule Evolution (RE). The SC procedure iteratively calls the RE procedure until all (or almost all) training instances (antigens) are covered by the discovered rules. The RE procedure evolves classification rules (antibodies) that are used to classify the instances. The best antibody is added to the set of discovered rules.

An ensemble of LCN-based classifiers was proposed by Valentini [4], where each classifier gives the probability that an instance belongs to a class. A combination phase then estimates the consensual probability. In [21] and [22], this method was modified to modulate relationships between the predictions of the classes and their descendants.

A global-based method using Ant Colony Optimization (ACO) was proposed by Otero et al. [23] to discover classification rules in the format IF …THEN …. The method uses a sequential instance-covering procedure to create rules that cover the majority of the instances. An empty set of rules is initialized, and rules are added to the list while the number of instances not covered by any rule is larger than a given threshold.

Cesa-Bianchi and Valentini [24] investigate the synergy between different LCN-based strategies applied to protein function prediction in the FunCat hierarchy. Kernel-based data fusion tools and ensemble algorithms were integrated with cost sensitive HMC methods [22, 25]. Synergy was defined as the improvement in the prediction accuracy, considering any evaluation measure, due to the use of concurrent learning strategies. Synergy is detected if the combination of two strategies achieves better correct classification rates than the average of the correct classification of the two strategies used individually [24].

The work of Stojanova et al. [26] reports a method which considers self-correlation in HMC, i.e., the statistical relationships between the same variable on different but related instances. The method is called Network Hierarchical Multi-label Classification (NHMC), and builds a generalized form of decision trees using the PCT framework, like Clus-HMC. During training, NHMC uses both the features of the instances, and the self-correlations between instances. The self-correlations are modeled as a network, which is exploited by the method during the learning phase.

Yu et al. [27] propose a method to predict protein function using incomplete hierarchical labels. The idea is to take the hierarchical and flat (non-hierarchical) similarities between functions and define a combined similarity between the labels. This similarity, together with the known labels, is used to estimate the missing functions of the proteins in the hierarchy. Afterwards, the method uses information about the interactions between proteins to predict their functions. In their study, the authors simulated the situation in which labels are missing in the hierarchy by randomly masking the leaf functions of a protein.

In this work, four methods reviewed in this section were used as baselines during the experimental analysis: the global decision tree based method Clus-HMC, and its local reductionist variants Clus-HSC and Clus-SC [8]; the Ant Colony Optimization based method *hm*Ant-Miner [23], which is a global method that achieved competitive results with Clus-HMC; and the method proposed by Stojanova et al. [26], which provides further information about the interaction among proteins.

These methods were chosen because they were evaluated using the same datasets we use. Also, they provide the same output format as HMC-LMLP, and the executables are freely available. Therefore, we were able to compare the prediction performance of these methods in detail.

The remainder sections of this paper are organized as follows. “Methods” Section presents the details of the new proposed HMC-LMLP variation, together with a brief description of its previous variants, and the methodology employed for the empirical analysis. The results are presented in “Results” Section, where the proposed method and its variants are compared with state-of-the-art methods for HMC on 10 protein function prediction datasets structured as trees. In “Discussion” Section we present the analysis and discussion on the results, and also perform an analysis to identify which functional combinations are predicted well and which ones are not. Finally, we summarize the conclusions and suggest topics for future work in “Conclusions” Section.

## Methods

The idea behind HMC-LMLP is to divide the learning process into a number of steps, aiming at learning a complex model through the combination of fewer simpler models, which are learned sequentially. This strategy is known in the ML literature as *reduction*, which converts a problem of minimizing a loss function into a problem of minimizing another, simpler loss function [7]. In our case, by reducing the problem, each model in sequence is forced to learn something different from the previously trained models, breaking down the complex learning process into simpler processes.

In HMC-LMLP, the reductionist approach works by learning MLP networks sequentially, one for each level of the class hierarchy. Each MLP is responsible for extracting local information from the instances at each level, which we believe to be useful in the classification of unlabeled instances. Since HMC problems are usually very complex, our hypothesis is that different patterns can be extracted from the instances in the different hierarchical levels. Whereas many different classification strategies could be employed in a similar architecture, we decided to use neural networks because of the simplicity in associating a class per output neuron. Therefore, obtaining a multi-label prediction for an instance is carried out in a straightforward fashion.

**Fig. 3:**
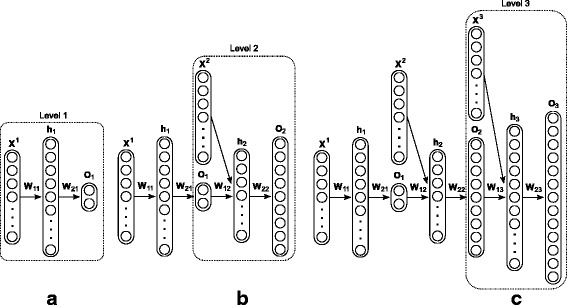
HMC-LMLP-Predicted architecture. **a** Training an MLP at the first level; **b** Using the output of the first MLP to augment the feature vector of the instances that are part of the training set of the MLP at the second level; **c** Using the output of the second MLP to augment the feature vector of the instances that are part of the training set of the MLP at the third level

Figure 3 illustrates the architecture of the HMC-LMLP method proposed in this work and its training process: **X**^*l*^ represents the instances assigned to classes from level *l*; **h**_*l*_ and **o**_*l*_ are, respectively, the hidden layer and output layer of the MLP network associated with level *l*; the matrices **W**_1*l*_ and **W**_2*l*_ represent, respectively, the weights connecting the input with the neurons in the hidden layer, and the neurons in the hidden layer with the output neurons of the MLP associated with level *l*.

Initially, an MLP is associated with the first hierarchical level. In order to allow the neural network to predict a set of labels, each output neuron is associated with one class. After the MLP has been trained for the first hierarchical level (Fig. 3a), a second MLP is associated with the next level of the hierarchy (Fig. 3b). The input for this network is now the instances feature vectors, augmented with the output provided by the previously trained MLP. Thus, each MLP from the second level onward uses the augmented feature vectors of those instances belonging to its respective associated level as inputs. The feature vectors that are used to train an MLP network at level *l* are augmented with the output from the MLP trained at level *l*−1.

The neural network associated with the first level is trained with all training instances (**X**^1^), since all instances are assigned to the classes from the first hierarchical level. At the second level, the MLP input is now the training instances that are assigned to the classes belonging to level 2 (**X**^2^), combined with the output provided by the previously trained MLP. The advantage of using the augmented feature vector for training each MLP is the incorporation of label dependency in the learning process. A similar approach was proposed in [28–30], where labels were used to augment the feature space of the instances in order to enable binary classifiers to discover existing label dependency by themselves.

The training of the neural network at the third level follows the same procedure adopted for the second level (Fig. 3c). This supervised incremental greedy procedure continues until the last level of the hierarchy is reached. Recall that when training an MLP network for level *l*, the neural network associated with level *l*−1 is used only to provide the inputs that will augment the feature vectors of the training instances for the MLP network associated with level *l*. MLPs associated with previous levels are not re-trained, because their training has already occurred in the previous steps.

For convenience, from now on the new version proposed here will be referred as HMC-LMLP-Predicted, considering that it employs the classes predicted by an MLP in one level to complement the feature vector of the instances that are part of the training set of the MLP in the subsequent level.

Also for convenience, from now on when we want to refer to all HMC-LMLP variants at the same time, we are going to use only the term HMC-LMLP.

Algorithms 1 and 2 show the pseudocodes for the HMC-LMLP-Predicted training and testing procedures. In Algorithm 1, *φ* and *φ*^′^ represent, respectively, the sigmoidal logistic activation function and its derivative regarding the argument. The gradients are represented by ***δ***, and *Δ* is used to denote the update to be applied to the synaptic weights.



### HMC-LMLP variants

Here we briefly present the other three previously proposed variants of HMC-LMLP, which will be considered baseline methods in the experiments. The very preliminary version [6] will be henceforth named HMC-LMLP-Labels, since it makes use of the classes predicted in one level as the single input to the MLP responsible for the predictions in the subsequent level. Thus, the instances feature vectors are used only to train the MLP associated to the first level.

The difference between HMC-LMLP-Predicted and HMC-LMLP-Labels, although subtle, is very important. From the second level onward, HMC-LMLP-Labels does not consider the instances attributes anymore. Only the predictions made at the previous level are used as input to the current MLP. Thus, there is no complementation of the feature vectors.

The other variants [10] will be called HMC-LMLP-True and HMC-LMLP-NoLabels. The first one employs, at each level, instead of predictions, the true labels of the instances from the previous level to complement the feature vectors. To treat HMC-LMLP-True as a baseline may sound counterintuitive, since using true labels seems to be better than using predictions. However, the idea here is to verify whether the neural networks are capable of detecting the class relationships by themselves, and verifying if the knowledge learned by an MLP can be useful in the training of the subsequent MLP. This idea was previously applied in the context of non-hierarchical multi-label classification [28]. In HMC-LMLP-True, on the other hand, the neural networks do not learn the class relationships by themselves, since they are fed a priori with the training class labels.

The HMC-LMLP-NoLabels variant uses only the original feature vectors to train the MLP at each level. Thus, an individual MLP is trained for each hierarchical level without using neither true nor predicted class labels to augment the feature vectors.

### Computing final predictions

In the test phase of HMC-LMLP-Predicted and HMC-LMLP-True (i.e., when predicting a test instance), a top-down strategy is employed. The test instance is given as input to the MLP associated to the first level, and the output from this MLP is used to augment the feature vector^1^. This augmented feature vector is then used as input to the MLP at the second level, whose prediction values will, once again, augment the input for the MLP at the third level. This procedure continues until the last MLP is reached. As previously mentioned, in both the training and test phases of HMC-LMLP-Predicted, the augmentation of feature vectors is non-incremental, i.e., the feature vector being fed into an MLP associated with level *l* is only augmented by the output from the MLP associated with level *l*−1. The same is true for HMC-LMLP-True, with the difference that the true class labels are used in the training phase and the predicted classes are used in the test phase. In HMC-LMLP-NoLabels, each MLP associated to each level is fed with the instances classified in classes belonging to the level. Each MLP then gives independent predictions for the instances at each level. In HMC-LMLP-Labels, only the MLP associated with the first level is fed with instances. From the second level onwards, each MLP is fed only with the output provided by the previous MLP.

To compute the final prediction for a test instance – considering all HMC-LMLP variants – we apply thresholds to the output prediction values from each MLP to define the predictions for each level. If the output of a neuron *j* is equal to or larger than a threshold, the instance is classified in class *c*_*j*_. As final classification, HMC-LMLP outputs a binary vector **v** of size |*C*|, where *C* is the set of all classes. If the output value of neuron *j* is equal to or larger than a given threshold, the value 1 is assigned to position **v**_*j*_. Otherwise, the position is set to 0. Different threshold values result in different predicted classes. Because we use the logistic sigmoid function as activation function in the neurons, the output values range from 0 to 1. We can then use threshold values in the interval [0,1]. Larger threshold values lead to small number of classes predicted, and smaller threshold values lead to higher number of classes predicted. During the classification process, the output values that are passed from network to network are not the values obtained after the application of a threshold (0 or 1). The regular output values from the previous-layer neurons, which are within [0,1], are not modified. The application of the threshold is only performed to compute the final predictions.

Considering all variations, after HMC-LMLP has provided the final predictions, a post-processing phase is employed to correct eventual classification inconsistencies, i.e., when a subclass is predicted without its superclass. This may occur because each neural network makes its own predictions, and even though these individual MLPs make use of data from the previous levels, this does not guarantee that the superclasses of all predicted subclasses have also been predicted. This problem is intrinsic to the LCL strategy [3]. The post-processing guarantees that the final predictions are consistent with the hierarchy.

We use a very simple procedure to correct inconsistencies in the predictions. The post-processing phase simply removes those predictions that do not have predicted superclasses.



### Computational complexity

In HMC-LMLP-Predicted and HMC-LMLP-True, each MLP has a time complexity of $\mathcal {O}(W_{l})$, with *W*_*l*_ the number of weights and biases of the MLP at level *l*. Assume that *A* is the number of attributes in the dataset, *H*_*l*_ is the number of hidden neurons of the MLP at level *l*, and *O*_*l*_ is the number of output neurons of the MLP at level *l*. We can then define *W*_1_ as (*A*+1)×*H*_1_+(*H*_1_+1)×*O*_1_. From the second level onwards, *W*_*l*_ is defined as (*O*_*l*−1_+*A*+1)×*H*_*l*_+(*H*_*l*_+1)×*O*_*l*_. Thus, the training cost of each neural network at each level *l* in HMC-LMLP is then $\mathcal {O}(W_{l} \times m_{l} \times n)$, with *m*_*l*_ being the number of training instances assigned to classes belonging to level *l*, and *n* the number of training epochs.

In HMC-LMLP-NoLabels, the computational cost is naturally lower, since the class labels are not used to augment the feature vectors. For HMC-LMLP-Labels, the computational cost in the first level considers only the number of features of the data. From the second level onwards, only the number of classes is considered, since the classes are the unique input of the MLP networks.

### HMC literature methods

Besides the previous proposed variants, we compare HMC-LMLP-Predicted with the following HMC methods used for protein function prediction: PCT-based methods Clus-HMC, Clus-HSC, Clus-SC [8], and *hm*Ant-Miner [23]. These methods are briefly described next:

- Clus-HMC: global-based method that builds a single decision tree to cope with all classes simultaneously;
- Clus-HSC: LCN-based method that applies a top-down strategy to induce a decision tree for each hierarchical class considering the hierarchical relationships;
- Clus-SC: LCN-based method that induces one decision tree for each hierarchical class without considering hierarchical relationships;
- *hm*Ant-Miner: global-based method that uses concepts from ACO to generate hierarchical multi-label classification rules.

Besides the aforementioned methods, we also compared our results with those provided by the method of Stojanova et al. [26], namely NHMC (Network Clus-HMC). Differently from the other methods, NHMC considers the interaction among proteins. This interaction is calculated based on the class label vectors associated to each protein. NHMC is built within the Clus-HMC framework, and also trains only one decision tree to cope with all classes simultaneously.

### Datasets

In the experiments, we used ten freely available^2^ protein function prediction datasets. The attributes of the datasets are related to issues like phenotype data and gene expression levels. The function are organized in a tree, according to the Funcat taxonomy.

The datasets are divided in subsets: training, validation and testing. Table 1 [6] presents their main characteristics, regarding to number of classes and instances. We present a brief description below, but more detailed biological description of each dataset can be found in [8] and in the corresponding references.

- **1 - Seq**: has attributes related to statistics obtained directly from the amino acid sequences, such as amino acid rates, sequence length and molecular weight. The atributes are mostly real value numbers, and were obtained using ProtParam [31] or taken from MIPS [32];

**Table 1:** Summary of datasets: number of attributes (|*A*|), number of classes (|*C*|), number of classes per level (Classes per level), total number of instances (Total) and number of multi-label instances (Multi)

Dataset	|*A*|	|*C*|	Classes per level	Training		Valid		Test	
				Total	Multi	Total	Multi	Total	Multi
1 - Seq [44]	478	499	18/80/178/142/77/4	1701	1344	879	679	1339	1079
2 - Pheno [44]	69	455	18/74/165/129/65/4	656	537	353	283	582	480
3 - Cellcycle [45]	77	499	18/80/178/142/77/4	1628	1323	848	673	1281	1059
4 - Church [46]	27	499	18/80/178/142/77/4	1630	1322	844	670	1281	1057
5 - Derisi [47]	63	499	18/80/178/142/77/4	1608	1309	842	671	1275	1055
6 - Eisen [48]	79	461	18/76/165/131/67/4	1058	900	529	441	837	719
7 - Expr [44]	551	499	18/80/178/142/77/4	1639	1328	849	674	1291	1064
8 - Gasch1 [49]	173	499	18/80/178/142/77/4	1634	1325	846	672	1284	1059
9 - Gasch2 [50]	52	499	18/80/178/142/77/4	1639	1328	849	674	1291	1064
10 - Spo [51]	80	499	18/80/178/142/77/4	1600	1301	837	666	1266	1047
- **2 - Pheno**: has attributes related to phenotypical data. They represent knock-out mutants missing in the sequence, regarding their growth or lack of growth. The data was obtained from databases such as MIPS [32] and TRIPLES [33]. The attribute values are all discrete, and the dataset is sparse;
- **3 to 10**: has real value attributes obtained using microarray chips to test the expression levels of genes across genomes [8];

We performed a pre-processing step before running HMC-LMLP over these datasets. We used the one-attribute-per-value strategy to convert all nominal attribute values into numeric values. In this strategy, an attribute with *k* categories is transformed into *k* binary attributes. In this study, we used −1 (absence) and 1 (presence) for each binary attribute. These are more suited for training neural networks [34]. The attributes were then standardized (mean 0 and variance 1). Additionally, all missing values for nominal and numeric attributes were replaced, respectively, by their mode and mean values.

### Evaluation method

As discussed in “Methods” Section, the outputs of HMC-LMLP for each class are real values in the interval [0,1], which is also true for the literature methods. Thus, a threshold value was employed to compute the final predictions from all methods. For the classification of an instance, if the output value for a given class is equal to or larger than the threshold, the instance is assigned to the class. Otherwise, it is not.

To choose an “optimal” threshold value is difficult, because low values lead to many predictions to each instance, resulting in high recall and low precision. On contrary, large values result in very few predictions, leading to high precision and low recall. Some studies try to find the “optimal” threshold value by modeling a threshold function as a linear function [35]. Others try to tune the threshold value by optimizing a given evaluation measure, or searching for the global maximum of the evaluation measure by using an optimization strategy [36].

In this work, we dealt with the problem of choosing a threshold by using precision-recall curves (PR-curves) [37]. To produce a PR-curve for a classification method, thresholds in the interval [0,1] are applied to their outputs, resulting in different values of precision and recall (point within the PR space), one for each threshold used. The union of these points form a PR-curve, and the area under the curve is calculated. Different methods can be compared based on their areas under the PR-curves.

The calculation of the area under the PR-curve is performed by the interpolation of the precision-recall points (PR-points) [37], and posterior connection. If we just connect the points without interpolation, the area below the curve would be artificially increased. Here, we used three variations of PR-curve: the area under the average PR-curve ($AU(\overline {PRC})$) and the weighted average of the areas under the individual (per class) PR-curves ($\overline {AUPRC}_{w}$).

The definitions of $AU(\overline {PRC})$ and $\overline {AUPRC}_{w}$ are given below [8]. The values of measures are in the interval [0,1]. The index *i*, in these equations, ranges from 1 to |*C*|. The number of true positives, false positives, and false negatives, are represented, respectively, by TP, FP, and FN.

#### Area under the average PR-curve

After applying a threshold, a PR-point ($\overline {Prec},\overline {Rec}$) is obtained through Eqs. (1) and (2). These equations are the micro-average of precision and recall.

1 $$ \overline{Prec} = \frac{\sum_{i} TP_{i}}{\sum_{i} TP_{i} + \sum_{i} FP_{i}}   $$

2 $$ \overline{Rec} = \frac{\sum_{i} TP_{i}}{\sum_{i} TP_{i} + \sum_{i} FN_{i}}   $$

#### Weighted average of the areas under the individual PR curves

We can obtain the weighted average of the areas under each PR-curve for each class separately. After calculating the *AUPRC*_*i*_ for each class, we compute the $\overline {AUPRC}_{w}$ through Eq. (3).

3 $$ \overline{AUPRC}_{w} = \sum_{i} w_{i} \times AUPRC_{i}   $$

In Eq. (3), we use *w*_*i*_ to weight the contribution of each class given its frequency, i.e., $w_{i} = v_{i}/\sum _{j} v_{j}$, with *v*_*i*_ the *c*_*i*_’s frequency in the dataset [8]. We also set all weights to be equal to 1/|*C*|. In this case, we refer to the measure as $\overline {AUPRC}$.

In hierarchical classification, it is important to give different weights to classes according to their level in the hierarchy. In protein function prediction, nodes located at deeper levels represent more specific protein functions, and thus are more frequent in the dataset. Nodes located at higher levels represent more general functions, thus being less frequent. It is then reasonable to consider that more frequent classes are more important depending on the application.

The significance of the results was verified using the non-parametric Friedman and Nemenyi statistical tests, more suitable when comparing many classifiers using several datasets [38]. The confidence level of 95 % was adopted. As in [8, 23], 2/3 of each dataset were used to train the classifiers (1/3 for training and 1/3 for validation), and 1/3 for test. We used exactly the same partition provided by Vens et al. 2008 [8].

### Parameters

We investigate the performance of HMC-LMLP using the conventional Back-propagation algorithm [39]. The HMC-LMLP parameters were optimized using the Eisen validation dataset. This dataset was selected because it was one of the datasets where Clus-HMC and Clus-HMC-ENS achieved their best performances, and also because it has a relatively small number of attributes, which makes it possible to run several experiments in a reasonable amount of time without feature selection. The following parameters were optimized:

- (i) number of neurons in each hidden layer. We considered all MLPs, from the one associated to the first level, to the one associated to the last level;
- (ii) parameters of the Backpropagation algorithm: learning rate and momentum constant;
- (iii) initial values of the neural network’s weights.

The number of hidden neurons of the MLPs associated to each level was gradually decreased as the corresponding level becomes deeper. This was performed to avoid overfitting, because as we go deep in the hierarchy, the number of training instances becomes smaller. Also, we try to reduce parameter selection influence by setting the number of hidden neurons as a fraction of the number of attributes used as input. We used the validation dataset to execute HMC-LMLP with different values for each of the parameters. We could not use all sets of values because of the large number of possibilities.

For the initial weights of the neural networks, parameter optimization showed higher initial values increased the chance of overfitting, resulting in a better performance on frequent classes but a worse overall prediction performance. The initial weights were varied by randomly selecting them initially from [−0.1,+0.1], but gradually increasing the range to [−1,1]. Regarding the number of neurons, a limited number of neurons for each hidden layer was tested. We gradually decreased these number from 1.0/0.9/0.8/0.7/0.6/0.5 neurons in each layer until 0.1/0.08/0.06/0.04/0.03/0.02. These numbers represent the fraction of the number of network attribute inputs. Thus, if a MLP has 100 inputs, 0.6 means that it has 60 hidden neurons.

We started our experiments with the same values used in the Weka machine learning toolkit [40] for learning rate and momentum. The learning rate is set to 0.3 and the momentum to 0.2. Gradually decreasing these values, we noticed that the neural networks became less prone to overfitting as these values decreased. The final parameters obtained for HMC-LMLP after the preliminary experiments are listed next.

- Number of hidden neurons in each level (fraction of the number of attribute inputs): 0.6/0.5/0.4/0.3/0.2/0.1;
- Backpropagation learning rate and momentum constant for the hidden and output layers: {0.05,0.03} and {0.03,0.01}, respectively;
- MLPs initial weights: within [−0.1,+0.1];

## Results

This section presents the experiments that were carried out to compare the prediction performance of the HMC-LMLP-Predicted with its previous variants [6, 10] and the literature HMC algorithms, namely, Clus-HMC, Clus-HSC, Clus-SC, and *hm*Ant-Miner. We compared those methods based on their overall prediction performance in all datasets, and also compared their performance in specific classes and levels of selected datasets. In the tables showing results, we refer to the HMC-LMLP variants as Labels, True and NoLabels (our previous versions), and Predicted (our new proposed version). Besides the evaluation measures, we also performed an analysis to verify which protein functions are well predicted by HMC-LMLP-Predicted, and also identify which ones are not.

In the experiments using HMC-LMLP, the results are the mean and standard deviation over 10 executions, each with randomly initialized weights. Given that *hm*Ant-Miner is a stochastic method, we also executed it 10 times and show the mean and standard deviation over all executions. Clus-HMC, Clus-HSC, and Clus-SC are deterministic algorithms and thus need to be executed only once.

When training HMC-LMLP, at each epoch we calculated its $AU(\overline {PRC})$ for the validation dataset. When this value stops increasing for 10 epochs, we stopped the training process and tested the best neural networks in the test dataset.

In addition, we also compared our results with the results provided by Network Clus-HMC (NHMC), a Clus-HMC variation proposed by Stojanova et al. [26]. This variation considers the protein features and additionally uses protein-protein interaction networks.

### Overall comparisons

Table 2 presents the $AU(\overline {PRC})$, $\overline {AUPRC}_{w}$ and $\overline {AUPRC}$ values for all methods that were compared. We highlight in bold the best results that were obtained.

**Table 2:** $AU(\overline {PRC})$, $\overline {AUPRC}_{w}$ and $\overline {AUPRC}$ values

	HMC-LMLP							
Dataset	Labels	Predicted	True	NoLabels	Clus-HMC	Clus-HSC	Clus-SC	*hm*Ant-Miner
$AU(\overline {PRC})$ values								
Cellcycle	0.185	**0.207**	0.203	0.205	0.172	0.111	0.106	0.155
Church	0.164	**0.173**	0.167	0.169	0.170	0.131	0.128	0.165
Derisi	0.171	**0.183**	0.176	0.182	0.175	0.094	0.089	0.149
Eisen	0.208	**0.245**	0.236	0.240	0.204	0.127	0.132	0.181
Gasch1	0.196	**0.236**	0.229	0.234	0.205	0.106	0.104	0.173
Gasch2	0.184	**0.211**	0.201	0.208	0.195	0.121	0.119	0.152
Pheno	0.159	0.159	0.158	0.159	0.160	0.152	0.149	**0.161**
Spo	0.172	**0.186**	0.180	0.184	**0.186**	0.103	0.098	0.177
Expr	0.196	**0.243**	0.238	0.240	0.210	0.127	0.123	0.180
Seq	0.195	**0.236**	0.233	0.232	0.211	0.091	0.095	0.186
$\overline {AUPRC}_{w}$ values								
Cellcycle	0.145	**0.184**	0.178	0.181	0.142	0.146	0.146	0.133
Church	0.118	**0.131**	0.129	0.127	0.129	0.127	0.128	0.123
Derisi	0.127	**0.146**	0.141	0.144	0.137	0.125	0.122	0.132
Eisen	0.163	**0.221**	0.210	0.213	0.183	0.169	0.173	0.151
Gasch1	0.157	**0.213**	0.207	0.211	0.176	0.154	0.153	0.154
Gasch2	0.142	**0.185**	0.174	0.179	0.156	0.148	0.147	0.142
Pheno	0.114	0.125	0.118	0.123	0.124	0.125	**0.127**	0.121
Spo	0.129	0.152	0.148	0.150	**0.153**	0.139	0.139	0.139
Expr	0.167	**0.236**	0.232	0.233	0.179	0.167	0.167	0.159
Seq	0.166	**0.220**	0.218	0.219	0.183	0.151	0.154	0.155
$\overline {AUPRC}$ values								
Cellcycle	0.022	0.035	0.031	0.033	0.034	0.036	**0.038**	0.030
Church	0.019	0.023	0.022	0.022	0.029	0.029	**0.031**	0.026
Derisi	0.020	0.027	0.024	0.025	**0.033**	0.029	0.028	0.031
Eisen	0.027	0.048	0.041	0.043	**0.052**	**0.052**	0.055	0.039
Gasch1	0.024	0.046	0.041	0.045	0.049	**0.047**	0.047	0.036
Gasch2	0.022	0.038	0.031	0.033	0.039	**0.042**	0.037	0.032
Pheno	0.019	0.023	0.021	0.022	0.030	**0.031**	**0.031**	0.028
Spo	0.021	0.027	0.026	0.026	0.035	**0.038**	0.034	0.032
Expr	0.021	0.053	0.051	0.051	0.052	**0.054**	0.050	0.038
Seq	0.019	0.041	0.041	0.041	**0.053**	0.043	0.042	0.036

Best results are highlighted in bold face

Table 3 shows the average rankings according to the Friedman test. The *p*-values obtained considering the $AU(\overline {PRC})$, $\overline {AUPRC}_{w}$, and $\overline {AUPRC}$ measures were, respectively, 1.47×10^−22^, 1.57×10^−12^, and 8.40×10^−18^, which clearly indicate that there are statistically significant differences among the methods. To identify which pairwise comparisons present statistically significant differences, we performed the Nemenyi post-hoc test.

**Table 3:** Average rankings according to the Friedman statistical test

Method	$AU(\overline {PRC})$	Method	$\overline {AUPRC}_{w}$	Method	$\overline {AUPRC}$
Predicted	1.35	Predicted	1.25	Clus-HSC	1.90
NoLabels	2.50	NoLabels	2.75	Clus-HMC	2.20
True	3.40	True	3.45	Clus-SC	2.70
Clus-HMC	3.55	Clus-HMC	3.85	Predicted	4.10
Labels	4.90	Clus-SC	5.55	NoLabels	5.45
*hm*Ant-Miner	5.30	Clus-HSC	5.70	*hm*Ant-Miner	5.50
Clus-HSC	7.20	Labels	6.65	True	6.15
Clus-SC	7.80	*hm*Ant-Miner	6.80	Labels	8.00

According to Nemenyi test, HMC-LMLP-Predicted outperformed Clus-HSC, Clus-SC, *hm*Ant-Miner, and HMC-LMLP-Labels with statistical significance considering both the $AU(\overline {PRC})$ and $\overline {AUPRC}_{w}$ measures. No statistically significant differences were detected between the HMC-LMLP variants and Clus-HMC. Considering the $\overline {AUPRC}$ measure, no statistically significant differences were detected among HMC-LMLP-Predicted and the other methods.

**Fig. 4:**
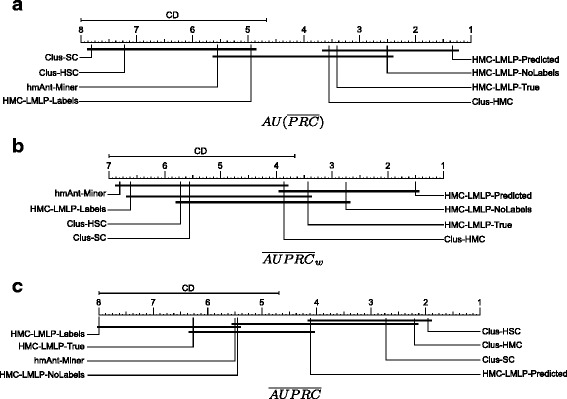
Results of the Nemenyi statistical test

The critic diagrams presented in Fig. 4 show the Nemenyi test results for the pairwise comparisons of all classifiers. In this kind of diagram, we connect the methods where no statistically significant results were detected. The $\overline {AUPRC}$ measure seemed to favor the PCT local-based methods, since Clus-HSC was statistically superior to HMC-LMLP-Labels, HMC-LMLP-True, HMC-LMLP-NoLabels and *hm*Ant-Miner. Recall that this measure considers all classes equally important.

**Fig. 5:**
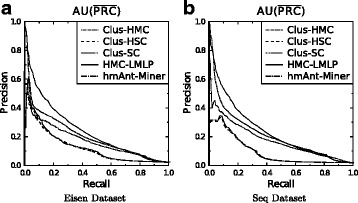
PR-curves of HMC-LMLP-Predicted, Clus-HMC, CLus-HSC, Clus-SC, and *hm*Ant-Miner

Figure 5 shows the PR-curves generated by HMC-LMLP-Predicted and the literature methods for the Eisen and Seq datasets, considering the $AU(\overline {PRC})$ measure. Regarding HMC-LMLP-Predicted and *hm*Ant-Miner, these curves were obtained for the best results in the validation dataset. Note that HMC-LMLP-Predicted provided the largest area under the curve in both datasets, when compared to the curves obtained by all methods. In many points of the curves, for a same recall value, HMC-LMLP-Predicted provided the highest precision values.

### Comparisons considering specific classes and levels

In this Section, we compared the HMC methods considering specific classes of the hierarchy, and also evaluate the methods level by level. We do so in order to examine their behavior when predicting classes in different hierarchical levels. We perform two sets of comparisons. First, we compared the methods to verify whether the use of the predictions to augment the feature vectors improved the results in specific classes and levels. For this purpose, we selected the Eisen dataset, which is the dataset where HMC-LMLP-NoLabels achieved its best classification results (Table 2) considering the $AU(\overline {PRC})$ measure. We selected, for each level, the three classes where HMC-LMLP-NoLabels achieved its best results, going down until the fourth level because in deeper levels the classes have a very low frequency in the training dataset (less than 5 %). The results for the Eisen dataset are shown in Table 4. The best absolute values are highlighted in bold. We did not consider the classes from the first level because the HMC-LMLP variants are different from each other only from the second level onwards.

**Table 4:** Specific-classes *AUPRC* values and per-level $AU(\overline {PRC})$ for the Eisen dataset

		HMC-LMLP							
Level	Classes	Labels	Predicted	True	NoLabels	Clus-HMC	Clus-HSC	Clus-SC	*hm*Ant-Miner
*AUPRC* values in specific classes									
2	12.01	0.198	0.760	0.744	**0.761**	0.582	0.655	0.651	0.565
2	10.01	0.164	**0.359**	0.348	0.357	0.217	0.174	0.220	0.232
2	11.02	0.265	**0.347**	0.325	0.337	0.248	0.247	0.266	0.221
3	12.01.01	0.173	**0.786**	0.771	0.779	0.609	0.724	0.706	0.570
3	11.02.03	0.239	**0.345**	0.314	0.330	0.236	0.244	0.215	0.212
3	14.13.01	0.054	**0.327**	0.255	0.247	0.177	0.123	0.159	0.153
4	11.02.03.04	0.201	**0.319**	0.294	0.294	0.208	0.203	0.230	0.172
4	10.01.09.05	0.063	**0.211**	0.190	0.192	0.123	0.103	0.150	0.101
4	11.02.03.01	0.089	0.128	**0.136**	0.121	0.091	0.085	0.130	0.092
Per-level $AU(\overline {PRC})$ values									
2	-	0.049	**0.113**	0.091	0.107	0.084	0.082	0.092	0.069
3	-	0.018	0.043	0.034	0.035	0.038	0.042	**0.049**	0.032
4	-	0.011	0.025	0.020	0.018	0.023	**0.035**	0.029	0.019
5	-	0.004	0.006	0.005	0.005	0.006	0.012	**0.013**	0.008
6	-	0.001	0.001	0.006	0.001	0.001	0.001	0.004	**0.006**

Best results are highlighted in bold face

In a second set of comparisons, we analyzed the results provided by HMC-LMLP-Predicted and the literature methods, i.e., Clus-HMC, Clus-HSC, Clus-SC, and *hm*Ant-Miner. As Clus-HMC is the state-of-the-art method so far, we performed the comparisons in the Seq dataset, in which Clus-HMC showed the best results considering the $AU(\overline {PRC})$ measure (Table 2). We selected, in each level, the three classes where Clus-HMC obtained its best results. We adopted the same procedure applied in the Eisen dataset, analyzing until the fourth level of the hierarchies when comparing performances in specific classes, but this time adding the results provided for the first hierarchical level. These results are presented in Table 5, with the best absolute values highlighted in bold.

**Table 5:** Specific-classes *AUPRC* values and per-level $AU(\overline {PRC})$ for the Seq dataset

Level	Classes	HMC-LMLP-Predicted	Clus-HMC	Clus-HSC	Clus-SC	*hm*Ant-Miner
*AUPRC* values in specific classes						
1	01	**0.589**	0.502	0.463	0.463	0.445
1	20	**0.546**	0.496	0.345	0.345	0.364
1	12	**0.573**	0.425	0.279	0.279	0.383
2	12/01	**0.697**	0.458	0.357	0.558	0.465
2	20/01	**0.495**	0.451	0.339	0.339	0.299
2	11/02	**0.401**	0.269	0.243	0.229	0.285
3	12/01/01	**0.769**	0.504	0.426	0.480	0.502
3	11/02/03	**0.400**	0.262	0.229	0.236	0.269
3	10/03/01	**0.196**	0.162	0.132	0.158	0.167
4	11/02/03/04	**0.385**	0.222	0.194	0.186	0.238
4	43/01/03/05	**0.168**	0.108	0.095	0.124	0.108
4	11/02/03/01	**0.132**	0.095	0.075	0.076	0.092
Per-level $AU(\overline {PRC})$ values						
1	-	**0.312**	0.246	0.224	0.224	0.234
2	-	**0.097**	0.070	0.062	0.071	0.063
3	-	0.033	0.033	**0.034**	0.032	0.027
4	-	0.015	0.017	**0.027**	0.026	0.016
5	-	0.005	0.008	**0.021**	0.014	0.008
6	-	0.012	0.002	0.010	**0.025**	0.006

Best results are highlighted in bold face

### Comparison with NHMC considering protein-protein interactions

Table 6 shows the results comparing all HMC-LMLP variations with NHMC, highlighting the best results. In Stojanova et al. [26], NHMC was evaluated using only the $\overline {AUPRC}$ measure. With NHMC, two protein-protein interaction networks were used together with the original dataset’s features: BioGRID [41] and DIP [42]. The BioGRID network contains physical and genetic interactions among proteins, while DIP (Database of Interacting Proteins) has information on binary protein-protein interactions, which are retrieved from research articles [26].

**Table 6:** $\overline {AUPRC}$ values for HMC-LMLP variations and NHMC

	HMC-LMLP				NHMC (DIP)		NHMC (Bio-GRID)	
Dataset	Labels	Predicted	True	NoLabels	*α*=0.5	*α*=0.0	*α*=0.5	*α*=0.0
$\overline {AUPRC}$ values								
Cellcycle	0.022	0.035	0.031	0.033	0.030	**0.037**	0.020	0.029
Church	0.019	**0.023**	0.022	0.022	0.020	0.020	0.020	0.020
Derisi	0.020	0.027	0.024	0.025	**0.028**	0.025	0.020	0.026
Eisen	0.027	**0.048**	0.041	0.043	0.042	0.025	0.024	0.037
Gasch1	0.024	**0.046**	0.041	0.045	0.040	0.032	0.033	0.036
Gasch2	0.022	**0.038**	0.031	0.033	0.034	0.027	0.025	0.028
Pheno	0.019	0.023	0.021	0.022	**0.035**	0.028	0.028	0.028
Spo	0.021	0.027	0.026	0.026	**0.029**	0.025	0.020	0.027
Expr	0.021	**0.053**	0.051	0.051	0.030	0.025	0.020	0.028
Seq	0.019	0.041	0.041	0.041	0.054	0.053	0.056	**0.062**

Best results are highlighted in bold face

Table 6 also shows two different results (*α*=0.5 and *α*=0.0) for both BioGRID and DIP. With *α*=0.0, NHMC considers only the protein-protein interactions to induce the decision tree. If *α*=0.5, NHMC equally weights variance reduction (Clus-HMC) and protein-protein interactions. For detailed information about Clus-HMC and NHMC implementations, the reader is referred to Vens et al. [8] and Stojanova et al. [26].

Stojanova et al. [26] also reported $AU(\overline {PRC})$ values obtained by NHMC in seven of the datasets used here. The reported values are the ones obtained using the DIP network with *α*=0.5. Table 7 shows the comparison of the HMC-LMLP results with their reported NHMC results.

**Table 7:** $AU(\overline {PRC})$ values of HMC-LMLP variations and NHMC

	HMC-LMLP				NHMC (DIP)
Dataset	Labels	Predicted	True	NoLabels	*α*=0.5
$AU(\overline {PRC})$ values					
Cellcycle	0.185	**0.207**	0.203	0.205	0.173
Church	0.164	**0.173**	0.167	0.169	0.152
Derisi	0.171	**0.183**	0.176	0.182	0.172
Eisen	0.208	**0.245**	0.236	0.240	0.196
Gasch2	0.184	**0.211**	0.201	0.208	0.186
Pheno	0.159	0.159	0.158	0.159	**0.241**
Spo	0.172	**0.186**	0.180	0.184	0.181

### Analysis regarding the predicted functions

Tables 8 and 9 present, respectively, the results for the best and worst predicted functions by HMC-LMLP-Predicted, together with the results obtained by Clus-HMC. By best predicted functions, we mean the functions where HMC-LMLP-Predicted obtained an *AUPRC* value higher than Clus-HMC in nine or more datasets. By worst predicted functions, we reported the functions where Clus-HMC performed better than HMC-LMLP-Predicted in nine or more datasets. In Tables 10 and 11, we give the descriptions of the these best and worst predicted functions. These descriptions were obtained from http://mips.helmholtz-muenchen.de/funcatDB/.

**Table 8:** Best predicted functions by HMC-LMLP-Predicted (means of *AUPRC* over the ten datasets)

Function	HMC-LMLP-Predicted	Clus-HMC		Function	HMC-LMLP-Predicted	Clus-HMC
01	0.493	0.421		14	0.373	0.319
01.01	0.219	0.087		14.13.01.01	0.146	0.092
01.03	0.094	0.061		16	0.292	0.260
01.05	0.269	0.199		16.01	0.148	0.109
02	0.305	0.214		16.19	0.082	0.056
10	0.389	0.316		20.01	0.239	0.216
10.01	0.214	0.165		20.01.01.01.01.01	0.146	0.004
10.01.05	0.126	0.085		30	0.075	0.061
10.01.05.01	0.104	0.058		32	0.234	0.181
10.03	0.272	0.219		32.01	0.226	0.162
10.03.02	0.107	0.063		34	0.150	0.131
10.03.01.01.03	0.012	0.010		34.11	0.107	0.083
11	0.418	0.344		34.11.03	0.095	0.068
11.02	0.274	0.216		41	0.021	0.013
11.02.03.01	0.108	0.075		42	0.280	0.250
11.04	0.204	0.143		43.01.03	0.169	0.129
11.04.01	0.222	0.126		43.01.03.05	0.122	0.093
12	0.528	0.410		–		
12.01	0.592	0.448		–		
12.01.01	0.612	0.463		–

**Table 9:** Worst predicted functions by HMC-LMLP-Predicted (means of *AUPRC* over the ten datasets)

Function	HMC-LMLP-Predicted	Clus-HMC		Function	HMC-LMLP-Predicted	Clus-HMC
01.01.03.01.01	0.0005	0.0008		01.05.05	0.0010	0.0137
01.01.03.03	0.0005	0.0032		01.05.05.04	0.0003	0.0006
01.01.03.05	0.0066	0.0096		01.05.05.07	0.0004	0.0069
01.01.03.05.02	0.0005	0.0009		01.20.05	0.0011	0.0015
01.01.05.01.01	0.0005	0.0009		01.20.05.09	0.0004	0.0009
01.01.06.01	0.0026	0.0042		01.20.17.03	0.0005	0.0009
01.01.06.01.01	0.0004	0.0008		01.20.19.05	0.0004	0.0008
01.01.06.01.02	0.0004	0.0008		01.20.31	0.0004	0.0007
01.01.06.04	0.0017	0.0039		02.01.01	0.0004	0.0009
01.01.06.04.01	0.0004	0.0007		02.16.03	0.0004	0.0007
01.01.06.04.02	0.0003	0.0006		02.16.11	0.0003	0.0006
01.01.09.01	0.0011	0.0073		16.06	0.0004	0.0007
01.01.09.01.02	0.0003	0.0067		20.03.02.02	0.0003	0.0031
01.01.09.04.01	0.0005	0.0009		20.09.07.29	0.0004	0.0009
01.01.09.05.01	0.0005	0.0009		30.05.01.10	0.0004	0.0009
01.01.11.01	0.0004	0.0009		32.07.05	0.0004	0.0009
01.01.11.02.02	0.0005	0.0009		34.07.02	0.0004	0.0008
01.01.11.03.02	0.0005	0.0009		38	0.0857	0.0710
01.01.11.04	0.0041	0.0073		38.07	0.0004	0.0008
01.01.11.04.02	0.0005	0.0009		40.01.03.01	0.0004	0.0007
01.02.02	0.0036	0.0041		40.10.02	0.0012	0.0015
01.02.02.09	0.0036	0.0041		40.10.02.02	0.0004	0.0009
01.02.02.09.01	0.0005	0.0009		40.10.02.02.01	0.0004	0.0009
01.02.02.09.05	0.0004	0.0009		–	–	–
01.02.07	0.0079	0.0158		–	–	–
01.02.07.03	0.0004	0.0008		–	–	–

**Table 10:** Best predicted functions by HMC-LMLP-Predicted

Function	Description
01	Metabolism
01.01	Amino acid metabolism
01.03	Nucleotide/nucleoside/nucleobase metabolism
01.05	C-compound and carbohydrate metabolism
02	Energy
10	Cell Cycle and DNA processing
10.01	DNA processing
10.01.05	DNA recombination and DNA repair
10.01.05.01	DNA repair
10.03	Cell cycle
10.03.02	Meiosis
10.03.01.01.03	G1/S transition of mitotic cell cycle
11	Transcription
11.02	RNA synthesis
11.02.03.01	General transcription activities
11.04	RNA processing
11.04.01	rRNA processing
12	Protein Synthesis
12.01	Ribosome biogenesis
12.01.01	Ribosomal proteins
14	Protein fate (folding, modification, destination)
14.13.01.01	Proteasomal degradation (ubiquitin/proteasomal pathway)
16	Protein with binding function or cofactor requirement (structural or catalytic)
16.01	Protein binding
16.19	Nucleotide/nucleoside/nucleobase binding
20.01	Transported compounds (substrates)
20.01.01.01.01.01	Siderophore-iron transport
30	Cellular communication/Signal transduction mechanism
32	Cell rescue, defense and virulence
32.01	Stress response
34	Interaction with the cellular environment
34.11	Cellular sensing and response to external stimulus
34.11.03	Chemoperception and response
41	Development (Systemic)
42	Biogenesis of cellular components
43.01.03	Fungal and other eukaryotic cell type differentiation
43.01.03.05	Budding, cell polarity and filament formation

**Table 11:** Worst predicted functions by HMC-LMLP-Predicted

Function	Description
01.01.03.01.01	Biosynthesis of glutamine
01.01.03.03	Metabolism of proline
01.01.03.05	Metabolism of arginine
01.01.03.05.02	Degradation of arginine
01.01.05.01.01	Biosynthesis of polyamines
01.01.06.01	Metabolism of aspartate
01.01.06.01.01	Biosynthesis of aspartate
01.01.06.01.02	Degradation of aspartate
01.01.06.04	Metabolism of threonine
01.01.06.04.01	Biosynthesis of threonine
01.01.06.04.02	Degradation of threonine
01.01.09.01	Metabolism of glycine
01.01.09.01.02	Degradation of glycine
01.01.09.04.01	Biosynthesis of phenylalanine
01.01.09.05.01	Biosynthesis of tyrosine
01.01.11.01	Metabolism of alanine
01.01.11.02.02	Degradation of isoleucine
01.01.11.03.02	Degradation of valine
01.01.11.04	Metabolism of leucine
01.01.11.04.02	Degradation of leucine
01.02.02	Nitrogen metabolism
01.02.02.09	Catabolism of nitrogenous compounds
01.02.02.09.01	Urea catabolism (not urea cycle)
01.02.02.09.05	Cyanate catabolism
01.02.07	Regulation of nitrogen, sulfur and selenium metabolism
01.02.07.03	Regulation of sulphur metabolism
01.05.05	C-1 compound metabolism
01.05.05.04	C-1 compound anabolism
01.05.05.07	C-1 compound catabolism
01.20.05	Biosynthesismetabolism of acetic acid derivatives
01.20.05.09	Biosynthesismetabolism of eicosanoids
01.20.17.03	Biosynthesismetabolism of amines
01.20.19.05	Biosynthesismetabolism of cobalamins
01.20.31	Biosynthesismetabolism of secondary products derived from L-lysine, L-arginine and L-histidine
02.01.01	Glycolysis methylglyoxal bypass
02.16.03	Lactate fermentation
02.16.11	Propionate fermentation
16.06	Motor proteinmotor protein binding
20.03.02.02	Symporter
20.09.07.29	Vesicle recycling
30.05.01.10	Two-component signal transduction system (sensor kinase component)
32.07.05	Detoxification by export
34.07.02	Cell-matrix adhesion
38	Transposable Elements, viral and plasmid proteins
38.07	Proteins necessary for the integration or inhibition of transposon movement
40.01.03.01	Regulation of directional cell growth
40.10.02	Apoptosis (type I programmed cell death)
40.10.02.02	Apoptotic program
40.10.02.02.01	Apoptotic mitochondrial changes

## Discussion

The results presented in Table 2 show that all HMC-LMLP variants outperformed the two local versions of the PCT-based methods Clus-HSC and Clus-SC by a large margin, considering the absolute values of the evaluation measures. The variants HMC-LMLP-Predicted, HMC-LMLP-True, and HMC-LMLP-NoLabels achieved better results than the global methods Clus-HMC and *hm*Ant-Miner for the vast majority of the datasets. Moreover, HMC-LMLP-Predicted improved the results achieved by versions HMC-LMLP-True and HMC-LMLP-NoLabels, which confirms that the predictions at one level were indeed useful in the learning process of the subsequent level.

It is interesting to see how the use of the predictions (HMC-LMLP-Predicted) instead of the true classes (HMC-LMLP-True) improved the algorithm’s classification performance. This is an indication that the neural networks were capable of better exploring the relationships between classes at each level when making use of the predictions, and that these relationships were learned during the training process.

The results shown in Table 2 also suggest that the variants HMC-LMLP-Predicted, HMC-LMLP-True, and HMC-LMLP-NoLabels achieved their best results in the most frequent classes in the hierarchy. As we can see, although the PCT-based methods performed better in the $\overline {AUPRC}$ measure, the performances of all methods were more similar in this measure, which gives equal importance to all classes. Given that the other two measures consider the frequencies of the classes in the datasets, the results suggest that HMC-LMLP performed better in the most frequent classes. For the $\overline {AUPRC}_{w}$ measure, the evaluation decreases the importance of the *AUPRC* values obtained in less frequent classes and increases the importance of more frequent classes.

According to the results from Table 4, the use of the predictions improved the classification performance in the majority of the classes. HMC-LMLP-Predicted also achieved the best correct classification rates when compared to the state-of-the-art methods. By analyzing the per-level $AU(\overline {PRC})$ values, all methods had a poor performance, specially from the third level onwards. Nevertheless, note that HMC-LMLP-Predicted outperformed the other HMC-LMLP variants.

Another feature that can be seen in Table 4 is that, from the third level onwards, the HMC-LMLP variants were outperformed (although by a very small margin) by the literature methods. This confirms the results observed in Table 2, where HMC-LMLP obtained the best $\overline {AUPRC}_{w}$ values, indicating that the best results were achieved in the most frequent classes. The very low frequency of the classes located at the deepest levels may explain the HMC-LMLP performance in these levels.

It is also possible to observe how much HMC-LMLP-Labels underperforms compared to the remaining methods, considering the deepest classes in the Eisen dataset. This behavior was expected, since HMC-LMLP-Labels does not employ the original attributes of the instances in the training process of the neural networks from the second level onwards, but only the predictions.

According to Table 5, HMC-LMLP-Predicted provided the best results in all analyzed classes. In the per-level evaluation, we can see that HMC-LMLP-Predicted obtained the best results in the top levels of the hierarchy, while the best performances in the deepest levels were obtained by the PCT-based methods. This is another evidence that HMC-LMLP performed better in the most frequent classes, as confirmed by its better overall results considering the $\overline {AUPRC}_{w}$ evaluation measure.

Considering the comparisons with Network Clus-HMC (Tables 6 and 7), HMC-LMLP-Predicted provided the best results in the majority of the cases. These results are particularly interesting given that our method makes use of features information only. We believe we could extend HMC-LMLP so it also considers protein-protein interactions, paving the way for a further increase in predictive performance.

Regarding the functions predicted, we can see by Table 8 that, for the best functions predicted by HMC-LMLP-Predicted, the differences between the *AUPRC* values obtained by HMC-LMLP-Predicted and Clus-HMC are much higher than the ones observed for the functions where Clus-HMC performed better than HMC-LMLP-Predicted (Table 9). This explains the best overall $AU(\overline {PRC})$ values obtained by HMC-LMLP-Predicted. Although Clus-HMC performed better in more classes, the individual *AUPRC* values for the classes where HMC-LMLP-Predicted performed better are much higher in favor of HMC-LMLP-Predited.

**Fig. 6:**
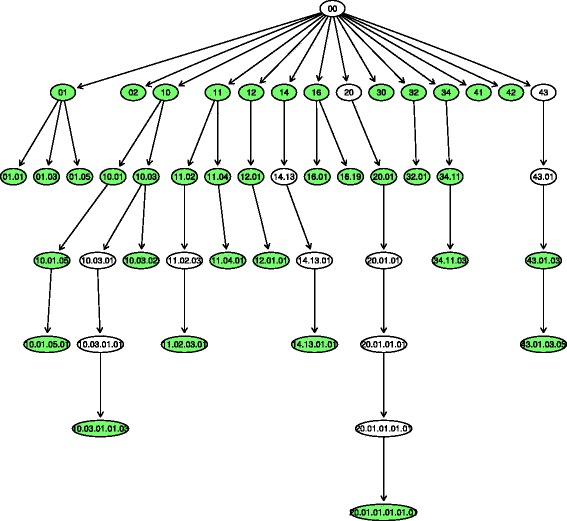
Subtree with best predicted functions by HMC-LMLP-Predicted in 90 % of the datasets

**Fig. 7:**
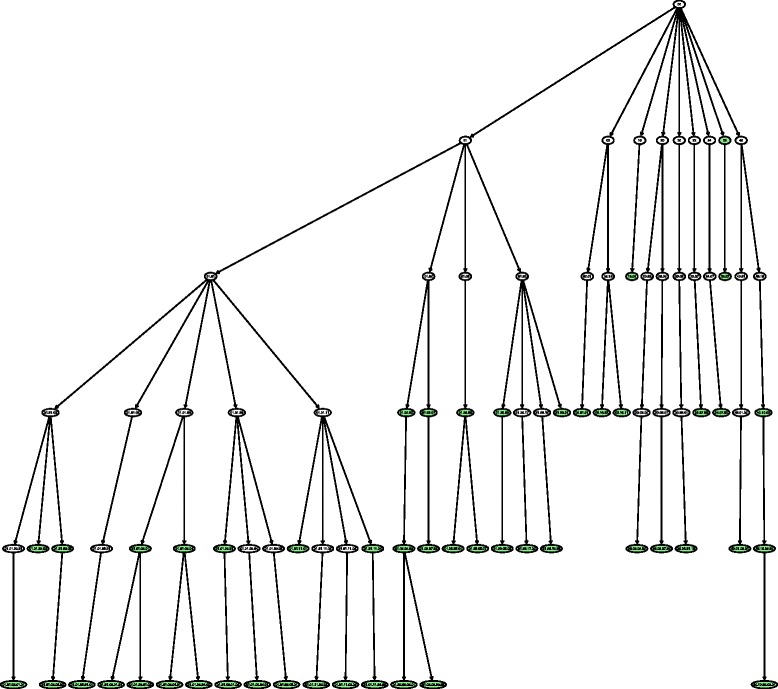
Subtree with worst predicted functions by HMC-LMLP-Predicted in 90 % of the datasets

In Figs. 6 and 7, we show the hierarchical positions of the best and worst predicted classes by HMC-LMLP-Predicted. These figures show the complete subtrees where the classes are located. We highlighted the classes shown in Tables 8 and 9, since the subtrees represent the transitive closure (all ancestor) of the classes presented in Tables 8 and 9.

By analyzing Fig. 6, we can have some insights about the prediction behavior of HMC-LMLP-Predicted. We can see that, in 90 % of the datasets investigated, it obtained best results than Clus-HMC mainly in the classes closer to the root. If we look at Fig. 7, we see that the functions where Clus-HMC performed better than HMC-LMLP-Predicted in 90 % of the datasets are mainly located at deeper levels. This confirms the results we’ve been observing so far.

**Fig. 8:**
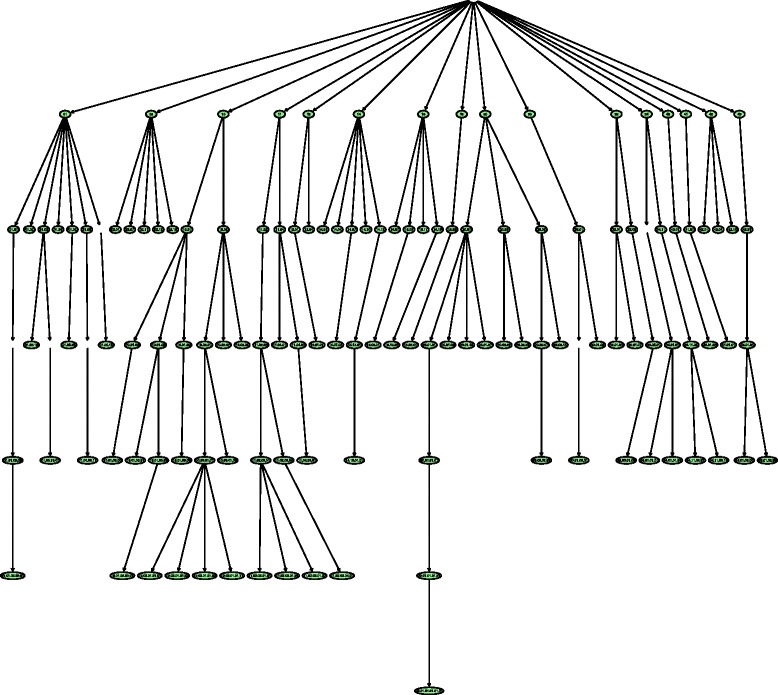
Subtree with best predicted functions by HMC-LMLP-Predicted in 60 % of the datasets

**Fig. 9:**
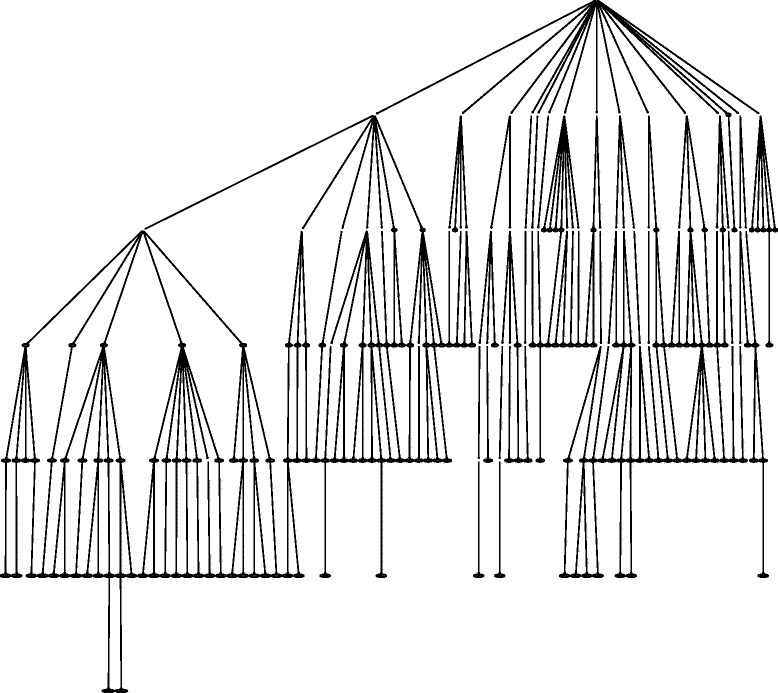
Subtree with worst predicted functions by HMC-LMLP-Predicted in 60 % of the datasets

We also performed an analysis considering the functions better predicted in six of the ten datasets. In Fig. 8, we show the subtrees with the functions where HMC-LMLP-Predicted obtained better *AUPRC* values than Clus-HMC. In Fig. 9, we present the functions where Clus-HMC performed better than HMC-LMLP-Predicted. We can see from these figures that the neural networks behavior remains the same, with the best predicted functions spread across the levels closer to the root, while the worst predicted functions more concentrated at the deepest levels, down until the fifth. Again, recall that Figs. 8 and 9 show the transitive closure of the best and worst HMC-LMLP-Predicted predicted functions. Thus, to improve visualization, we deleted the nodes which are not in the set of the best and worst predicted functions.

Another characteristic that can be observed in the figures showing the subtrees is that HMC-LMLP-Predicted obtained its best results in complete paths starting at the root node. For example the paths 10.01.05.01 and 12.01.01. The path 12.01.01, particularly, contains the classes where HMC-LMLP-Predicted obtained its best *AUPRC* values 0.528 (12), 0.592 (12.01) and 0.612 (12.01.01).

As we use the logistic function in the output neurons of each MLP, the outputs of the neurons associated to each class can be interpreted as probabilities of instances to belong to the corresponding class. As HMC-LMLP-Predicted obtained better results than Clus-HMC in classes closer to the root (more frequent classes), we can say that the neural networks provided stronger evidence about the pertinence of the proteins to the functions considered more important in the problem domain. Recall that both HMC-LMLP and Clus-HMC weights the classes during evaluation, considering more important the frequent classes.

Still, higher *AUPRC* values associated to a given class means that, for high thresholds applied to the output of the neurons, the precision and recall values remain high. This can be interpreted as a high reliability associated to the prediction given by the neuron.

Even though we validate the neural networks and evaluate their final classification predictive performance using $AU(\overline {PRC})$, this is not the loss function minimized during the network training. Each MLP is trained by minimizing the mean square error (MSE) of its corresponding layer. As previously mentioned, when an MLP is being trained and validated for level *l*, it minimizes the MSE only for the level *l*, even though the $AU(\overline {PRC})$ for the hierarchical classification considering the predictions made in every level of the hierarchy up to that point is being calculated. Despite reducing the HMC problem to several flat (non-hierarchical) multi-label problems, we are interested in reaching satisfactory precision and recall values within the original HMC problem. When training and validating an MLP at level *l*, a model is being induced for a hierarchy with *l* levels. In several neural networks applications, it is very common to minimize the MSE during training, whereas the classifier predictive performance is evaluated using another evaluation measure [35].

According to [7], when reducing a problem *y* to another problem *x*, a method to solve the problem *x* can solve the problem *y* using a transformation. In HMC-LMLP, we transform a hierarchical multi-label problem into *k* non-hierarchical multi-label problems, and minimize the MSE for each problem separately. Thus, we transform the original hierarchical multi-label distribution *D* into non-hierarchical multi-label distributions *D*^′^. If we consider *HMC*_*h*_ the hierarchical multi-label method proposed, and *h* the individual neural networks applied to each flat multi-label problem, the error obtained by *HMC*_*h*_ on *D* is bounded by the error obtained by *h* on *D*^′^, *i*.*e*., *e*(*HMC*_*h*_,*D*)≤(*k*−1)*e*(*h,D*^′^). In HMC problems, errors committed for a given level are propagated to deeper levels. Thus, the worst case error in *D* occurs when an *HMC*_*h*_ error committed in the first level is propagated to the the last level, which is a leaf. This is equivalent to summing up the individual *h* errors obtained in *D*^′^ for each level.

The HMC-LMLP variants estimate different quantities depending on the input used in the neural networks. The distributions are modified in each variant, modifying the input of the MLP at each level. For example, in HMC-LMLP-NoLabels, only the features are taken into account, resulting in the estimation of probabilities *P*(*y*|**x**), where *y* is a class of the hierarchy. The variants HMC-MLP-Predicted and HMC-LMLP-Labels also estimates *P*(*y*|**x**) because the predicted labels are functions of **x**. The HMC-MLP-Predicted variant uses both the original features and the specific functions of these features (predictions in previous level). On the other hand, HMC-LMLP-Labels strongly constrains the hypothesis space, because from the second level onwards it uses only functions of original labels. This is the reason this method performs poorly.

The variant HMC-MLP-True, in turn, estimates *P*(*y*|**x**,*y*^′^) probabilities, where *y*^′^ are the true class labels in the previous level. As we have previously observed in the empirical analysis, this difference between the variants lead to different results. Indeed, estimating *P*(*y*|**x**) resulted in an increased classification performance in the case of HMC-LMLP-Predicted. In both HMC-LMLP-Predicted and HMC-LMLP-True, the output from the previous level (predictions) are treated as new features. The difference between HMC-LMLP-Predicted and HMC-LMLP-True is that, in the former, these new features are real values [0,1], which are functions of **x**, while in the latter the new features are either 0 or 1, and not functions of **x** (predictions).

## Conclusions

In this study, we have proposed a new reduction strategy for hierarchical multi-label classification. We have presented a substantial extension of a previous method we proposed for hierarchical multi-label classification, namely Hierarchical Multi-Label Classification with Local Multi-Layer Perceptrons (HMC-LMLP), which trains a Multi-Layer Perceptron (MLP) per hierarchical level, with each MLP being responsible for the predictions in its associated level. The novel method, namely HMC-LMLP-Predicted, uses the predictions made in a given level to augment the feature vectors of all instances that are used in the training of the MLP for the subsequent level. Additionally, in order to verify whether the use of the predictions improved classification performance, we used two additional variants. The first variant makes use of the true classes to augment the feature vectors in each level (HMC-LMLP-True), and the second variant employs the input features alone, without any further augmentation (HMC-LMLP-NoLabels).

We performed several experiments using datasets. According to the experimental results, the newly proposed HMC-LMLP variant achieved the best classification results overall, when compared to different state-of-the-art methods from the literature. Besides, the new variant – HMC-LMLP-Predicted – improved the classification performance when compared with HMC-LMLP-True and HMC-LMLP-NoLabels. We identified which functions were better and worst predicted by our method, and demonstrated, by using two different variants of the area under the Precision-Recall Curves, that HMC-LMLP performs better for the most frequent classes of the hierarchies.

As future work, we intend to implement an ensemble of HMC-LMLP, and compare it with the Clus-HMC ensemble. Although neural networks have a higher computational cost, we believe that the use of GPU-based parallel computation techniques will speed up the HMC-LMLP training process, allowing for a fair comparison with ensembles of PCT. We also plan to use hierarchies structured as DAGs and to incorporate protein-protein interaction information during learning. Finally, we want to further investigate the impact of different strategies for solving the error-inconsistency problem within HMC-LMLP.

## Endnotes

^1^ Recall that, in the test phase, the true labels are not available to the MLPs.

^2^https://dtai.cs.kuleuven.be/clus/hmcdatasets/.
